# Degradation behavior and osseointegration of Mg–Zn–Ca screws in different bone regions of growing sheep: a pilot study

**DOI:** 10.1093/rb/rbac077

**Published:** 2022-10-18

**Authors:** Romy Marek, Hanna Ćwieka, Nicholas Donohue, Patrick Holweg, Julian Moosmann, Felix Beckmann, Iva Brcic, Uwe Yacine Schwarze, Kamila Iskhakova, Marwa Chaabane, Sandra Sefa, Berit Zeller-Plumhoff, Annelie-Martina Weinberg, Regine Willumeit-Römer, Nicole Gabriele Sommer

**Affiliations:** Department of Orthopaedics and Traumatology, Medical University of Graz, 8010 Graz, Austria; Institute of Metallic Biomaterials, Helmholtz-Zentrum Hereon GmbH, 21502 Geesthacht, Germany; National Institute for Bioprocessing Research and Training, University College Dublin, Dublin 4, Ireland; Department of Orthopaedics and Traumatology, Medical University of Graz, 8010 Graz, Austria; Institute of Materials Physics, Helmholtz-Zentrum Hereon GmbH, 21502 Geesthacht, Germany; Institute of Materials Physics, Helmholtz-Zentrum Hereon GmbH, 21502 Geesthacht, Germany; D&R Institute of Pathology, Medical University of Graz, 8010 Graz, Austria; Department of Orthopaedics and Traumatology, Medical University of Graz, 8010 Graz, Austria; Department of Dental Medicine and Oral Health, Medical University of Graz, 8010 Graz, Austria; Institute of Metallic Biomaterials, Helmholtz-Zentrum Hereon GmbH, 21502 Geesthacht, Germany; SCANCO Medical AG, 8306 Wangen-Brüttisellen, Switzerland; Institute of Metallic Biomaterials, Helmholtz-Zentrum Hereon GmbH, 21502 Geesthacht, Germany; Institute of Metallic Biomaterials, Helmholtz-Zentrum Hereon GmbH, 21502 Geesthacht, Germany; Department of Orthopaedics and Traumatology, Medical University of Graz, 8010 Graz, Austria; Institute of Metallic Biomaterials, Helmholtz-Zentrum Hereon GmbH, 21502 Geesthacht, Germany; Department of Orthopaedics and Traumatology, Medical University of Graz, 8010 Graz, Austria

**Keywords:** biodegradable implants, magnesium-based alloys, computed tomography, Mg–Zn–Ca, sheep, histology

## Abstract

Magnesium (Mg)-based implants are highly attractive for the orthopedic field and may replace titanium (Ti) as support for fracture healing. To determine the implant–bone interaction in different bony regions, we implanted Mg-based alloy ZX00 (Mg < 0.5 Zn < 0.5 Ca, in wt%) and Ti-screws into the distal epiphysis and distal metaphysis of sheep tibiae. The implant degradation and osseointegration were assessed *in vivo* and *ex vivo* after 4, 6 and 12 weeks, using a combination of clinical computed tomography, medium-resolution micro computed tomography (µCT) and high-resolution synchrotron radiation µCT (SRµCT). Implant volume loss, gas formation and bone growth were evaluated for both implantation sites and each bone region independently. Additionally, histological analysis of bone growth was performed on embedded hard-tissue samples. We demonstrate that in all cases, the degradation rate of ZX00-implants ranges between 0.23 and 0.75 mm/year. The highest degradation rates were found in the epiphysis. Bone-to-implant contact varied between the time points and bone types for both materials. Mostly, bone-volume-to-total-volume was higher around Ti-implants. However, we found an increased cortical thickness around the ZX00-screws when compared with the Ti-screws. Our results showed the suitability of ZX00-screws for implantation into the distal meta- and epiphysis.

## Graphical Abstract

**Figure:**
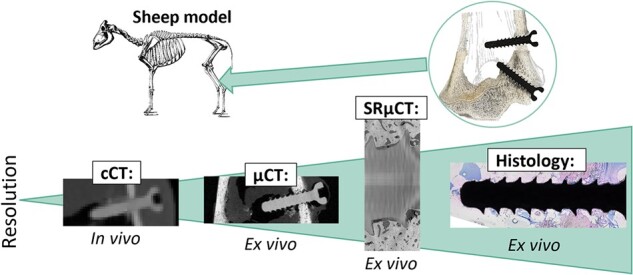

## Introduction

Commonly used metallic implants, such as titanium (Ti) or stainless steel, remain the clinician’s first choice in orthopedic and trauma surgery based on their mechanical properties and corrosion resistance. Especially in load-bearing indications, high strength is warranted to guarantee appropriate fracture healing by overtaking the load-bearing capacity of the bone. However, the mechanical mismatch between permanent implants and bone leads to stress shielding effects, resulting in bone loss or even secondary fracture over time [1]. In case of chronic pain and other complications, implant removal is frequent and accounts up to 80% in fractures treated with osteosynthesis [2]. Moreover, second removal surgery after bone fracture healing is recommended in children and adolescents, to avoid negative long-term reactions. To reduce these shortcomings, biodegradable metals can be used, where a great potential is indicated for magnesium (Mg)-based alloys. Mg alloys exhibit mechanical properties similar to those of human bone and excellent biocompatibility [3], thus reducing the stress-shielding effect [4]. Due to their biodegradability in the physiological environment, Mg implants do not require removal after fracture healing [1] and thus reduce pain, the chance of late infection by reduced immunological capacity in a long view as complications of permanent implants. However, strong hydrogen and ion release upon Mg degradation leads to local pH changes, loss of mechanical integrity and gas pocket accumulation in the vicinity [5]. In fracture treatments, the priority is stabilization of the broken bones without adverse effects of fracture consolidation. It is of utmost interest to control the degradation of a resorbable implant during fracture healing in a period of up to 12 weeks [6]. Recent pre-clinical *in vivo* studies introduced a new bioresorbable Mg–zinc–calcium alloy (ZX00: composition Mg < 0.5 Zn < 0.5 Ca in wt%) as a prospective material for pediatric trauma treatment. Grün *et al*. investigated the application of ZX00 in growing-rat and growing-sheep models and demonstrated homogeneous degradation of ZX00-implants with good biocompatibility and osseointegration [7]. Moreover, Holweg *et al*. [8] and Herber *et al*. [9] showed a good clinical outcome after operative treatment of medial malleolus fractures with ZX00-screws in humans. However, implant degradation is strongly influenced by the direct environment, such as tissue types of the peri-implant area. When implanting an orthopedic device into the bone tissue, it is exposed to blood and body fluid. Previous studies focused on the differences in degradation behavior between soft and hard tissue [10]. For further clinical applications in orthopedics and trauma surgery, it is of utmost interest to reveal bone-region-dependent degradation characteristics, to augment the portfolio of clinical use or to design the implant itself according to the degradation behavior. In this study, we evaluated differences in degradation behavior of ZX00-screws and corresponding osseointegration between distal metaphysis, physis and epiphysis. The metaphysis contains cortical bone structures and parts of the intramedullary cavity. The epiphysis contains only few cortical bone, but mainly trabecular bone structures. Furthermore, the physis is allocated in the epiphysis, which is responsible for the longitudinal growth of the bones [11]. The trabecular bone areas proximal and distal of the physis are supplied by two different artery branches and thus possess different perfusion profiles [12]. The physis itself is only poorly perfused. Therefore, we expected different degradation performances for each bone region.

To monitor the implant behavior during *in vivo* and *ex vivo* studies, we used non-destructive, 3D imaging techniques, specifically clinical computed tomography (cCT), micro computed tomography (µCT) and high-resolution synchrotron radiation micro computed tomography (SRµCT). The cCT images reveal low spatial resolution, but the low X-ray exposure allows measurements on living animals at different stages of healing. Thus, the dynamic evolution of bone growth and implant degradation can be assessed. For *ex vivo* experiments, we used µCT with higher spatial resolution for performing quantitative analysis of metal degradation. The entire implant was investigated in the distal meta- and epiphysis. To gain a more detailed insight into bone growth and degradation in the different bone types, smaller regions of interest were evaluated using high-resolution SRµCT measurements [13, 14] and histology [15].

## Materials and methods

### Ethical statement

The animal trial (Permit Number: BMWFW-66.010/0073-WF/V/3b/2015) was approved by the Austrian Federal Ministry for Science and Research and followed the guidelines on accommodation and care of animals formulated by the European Convention for the Protection of Vertebrate Animals Used for Experimental and Other Scientific Purposes under consideration of the 3R principles for animal welfare.

### Material production

Raw material production of ZX00 and extrusion of 6-mm rods was carried out at ETH Zürich in cooperation with de Cavis AG (Swiss Federal Laboratories for Materials Science and Technology, Dübendorf, Switzerland) as described by Holweg *et al*. [11].

### Implant fabrication

In order to avoid potential contamination and corrosive attack, polycrystalline diamond tools were used without lubrication for the screw manufacturing [11]. The screws were cleaned in an ultrasonic bath in acetone, air dried in a clean-room atmosphere and sterilized by gamma irradiation with a maximum dose of 29.2 kGy [11]. The ZX00-screws were manufactured by Wittner (Ernst Wittner GmbH, Wien, Austria) with an outer diameter of 3.5 mm and a length of 16 mm. Their initial volume calculated from µCT data of three screws scanned before implantation was 112.03 ± 5.42 mm^3^, with a surface area of 226.94 ± 9.67 mm^2^. Ti-screws of the alloy Ti6Al4V produced by Hofer (Hofer Medical Solutions, Fürstenfeld, Austria) possessed similar diameter with a length of 18 mm.

### Surgery and postoperative treatment

One 3-month-old lamb was implanted with ZX00- and Ti-screws for each observation period of 4, 6 and 12 weeks. All animals received pre-operative tetanus prophylaxis. Surgeries were performed under sterile conditions. Only ZX00-screws were used for the right leg. For the left leg, only Ti-screws were used, except for the ZX00-screws in the proximal epiphysis (Supplementary Fig. S1). For this study, only the screws in the distal metaphysis and epiphysis of both legs were considered. For these screws, two ∼2–3 cm long skin incisions were performed at the distal medial epiphysis and metaphysis, followed by precise epiperiosteal dissection to the bone under protection of the local neurovascular bundle. Drilling was performed with a 2.7-mm drill bit and a 3.5-mm thread tapper. The epiphyseal screw was inserted in a latero-cranial, and the metaphyseal screw in a lateral direction. Finally, the tissues were closed in layers, followed by disinfection using iodine solution. Postoperative treatment included analgesia with carprofen and buprenorphine for 4 days post-surgery, as well as infection prophylaxis with gentamicin and penicillin for 5 days. Wounds were checked daily for ∼1.5 weeks. One animal was euthanized after 4, 6 or 12 weeks, respectively. Tibiae were excised and cut into proximal part, shaft and distal part. Finally, they were wrapped in saline-soaked gauzes and frozen at –20°C until further analysis.

### Imaging and radiological analysis

#### Clinical computed tomography

*In vivo* low-resolution cCT was performed after 4, 6 and 12 weeks at the Department of Radiology at the Medical University of Graz, using a Siemens Sensation Cardiac 64 CT device (Siemens, Erlangen, Germany). The operating voltage was set to 120 kV and 35 mA, which results in a combined dose of 13.42 mGy and a resolution of 0.6 mm per voxel. Acquired data was qualitatively investigated using MIMICS^®^ software (version 21.0; Materialise, Leuven, Belgium).

#### Micro computed tomography

**Figure 1.**
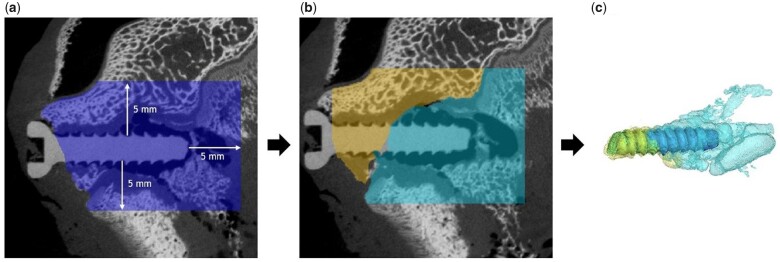
Segmentation of implant and gas volume from µCT data. Total volume (in blue), which was considered for gas evaluation in the epiphyseal area, in a distance of 5 mm to the implant (**a**). Gas evolution within the soft tissue was not considered. The total volume was further divided into Sub-ROIs. In the epiphysis, it was distinguished between trabecular bone proximal (in turquois) and trabecular bone distal (in yellow) of the physis (**b**). Segmentation of implant and gas volume was performed for each region (**c**).

*Ex vivo* µCT scans were performed at the Medical University of Graz using a Bruker Skyscan 1276 (Bruker, Germany). The distal parts of the tibiae, consisting of meta- and epiphysis were scanned in the µCT. Operating voltage and current of 100 kV and 200 µA were set, respectively, with rotation steps of 0.4°. Aluminum and copper filters with a thickness of 1 mm and 0.05 mm, respectively, were used. The binned pixel size was set at 20.3 µm. For the reconstruction of the acquired data, we used the software NRecon (Bruker, Germany). Three-dimensional post processing, segmentation and measurement of the μCT data sets were performed with MIMICS^®^ software (version 21.0; Materialise, Leuven, Belgium). The screw degradation was investigated by evaluating the degradation rate (DR) [mm/year] from the change in implant volume over time. Following modified equation from Nidadavolu *et al*. [16] was used for the calculation of the DR:

(1) $$DR=\frac{V_{i}-V_{r}}{A_{i}t}=\;\frac{VL}{A_{i}t}$$

where $V_{i}$ is the initial volume and $V_{r}$ the residual volume, which give the volume loss (VL; in mm^3^) when subtracted from each other. A_i_ is the initial surface area and *t* is the time of degradation. As the epiphyseal and metaphyseal screws were close to each other, distinguishing between the gas cavities of the respective screws was not feasible. Thus, gas evolution was evaluated within a defined region of interest (ROI), which was set to 5 mm from the screw surface within the bone area (Fig. 1a). Gas evolution within the soft tissue was not considered, as most soft tissue was removed prior to scanning. By this method, gas evolution within the ROIs in the distal epiphysis, as well as in the distal metaphysis was calculated.

**Figure 2.**
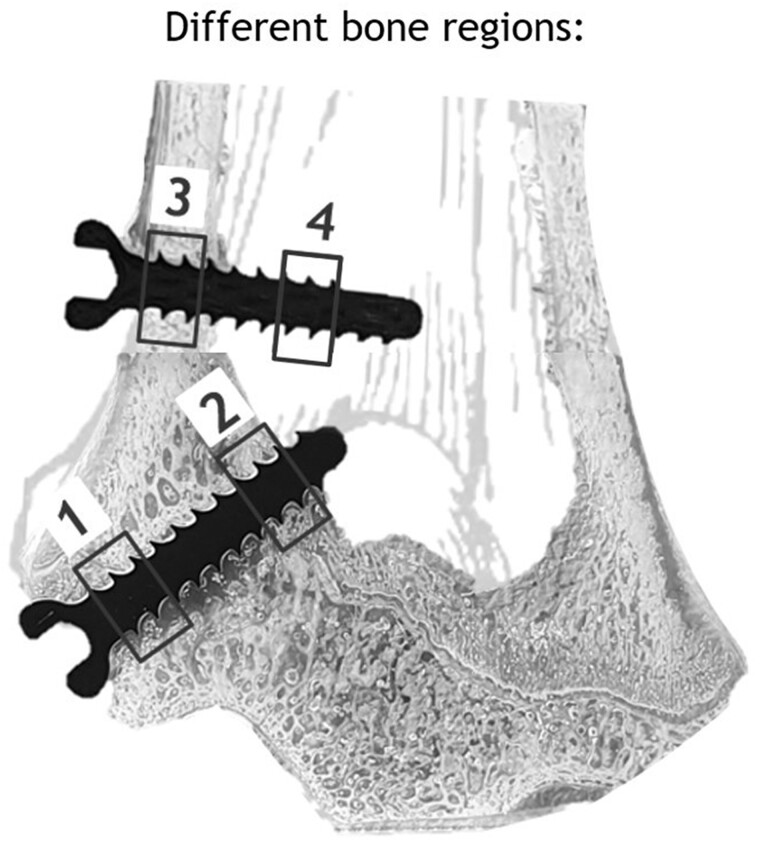
Distal epiphysis and metaphysis with four defined ROIs. 1: trabecular bone distal of physis (dpROI); 2: trabecular bone proximal of physis (ppROI); 3: cortical bone (cROI); and 4: intramedullary cavity (iROI).

To additionally investigate gas evolution in different bone types, the ROIs were further divided into the following four sub-ROIs: dpROI = trabecular bone distal of the physis; ppROI = trabecular bone proximal of the physis; cROI: cortical bone; and iROI: intramedullary cavity (Fig. 2). For each sub-ROI, content of implant and gas volume was evaluated (Fig. 1b and c). As these sub-ROIs possessed different volumes, we normalized each ROI to 100 mm³. Finally, for each normalized ROI and sub-ROI, containing implant and gas volume, as well as their ratio were calculated.

#### Synchrotron radiation micro computed tomography

SRµCT imaging was performed at the P07 high-energy material science beamline [17], which is operated by Hereon at the PETRA III storage ring at the Deutsches Elektronen-Synchrotron in Hamburg, Germany. Samples were scanned at a photon energy of 60 keV with the use of a double crystal monochromator. Due to the large sample size, two ROIs in specific sections of the screw were imaged instead of the whole volume. The sample was imaged by rotating off-center ∼360° leading to a vertical and horizontal field of view of 2.88 mm and 12.56 mm, respectively. Projections were stitched prior to tomographic reconstruction using IDL (Harris Geospatial Solutions, Inc.). The effective pixel size was 1.06 µm, which was binned during reconstruction to a pixel size of 3.18 µm. Stitched tomograms were reconstructed using a reconstruction framework implemented in MATLAB [18, 19] and employing the ASTRA toolbox for tomographic back projection [20, 21]. The reconstructed data sets were filtered using an iterative nonlocal means filter [22].

The segmentation of the filtered data sets was performed in Avizo 2021.1 (FEI SAS, Thermo Scientific, France). The labels residual material (non-corroded alloy), degradation layer (corrosion products attached to the residual material), bone (mineralized tissue) and background (all remaining features not assigned to previously mentioned labels) were distinguished. The segmented data were used for quantitative analysis of parameters describing degradation of implants, osseointegration and bone regeneration over the healing process [13]. Prior to the analysis of VL and DR, the alignment of non-degraded screw and ROIs of degraded screw were necessary. The mid-resolution µCT scan of a non-degraded implant was used as a reference shape and it was registered and resampled on the degraded ones. With this operation, we ensure that compared volumes are from the same sections of the screw.

The quantitative parameters were VL (in mm^3^), DR (in mm/year), bone-to-implant contact (BIC) [%] and bone volume fraction (BV/TV) [%]. The volumes of non-degraded and degraded screws as well as the volume of bone and background in selected areas were calculated in software Fiji (ImageJ) [23] based on segmented labels.

VL and DR quantifications were quantified according to equation (1).

The BIC parameter was used for characterizing the osseointegration and as an indication for implant stability in the bone:

(2) $$\mathrm{BIC}=\frac{\#\mathrm{boundary}\;\mathrm{voxels}\;\mathrm{of}\;\mathrm{implant}\;\mathrm{in}\;\mathrm{contact}\;\mathrm{with}\;\mathrm{bone}}{\#\mathrm{total}\;\mathrm{surface}\;\mathrm{voxels}\;\mathrm{of}\;\mathrm{implant}}$$

Both values were calculated with the use of a MATLAB R2018a (The MathWorks, Inc., USA) script which determined the contact voxels in the 3D volume between two different layers [24]—implant (degradation layer + residual metal or just material in case of Ti) and bone, and implant and background, respectively.

BV/TV is a parameter used for evaluating the bone formation surrounding an implant. A ROI was determined separately for each sample by enlarging the non-degraded, registered reference screw by 30 µm and 1 mm, respectively. The enlargement of 30 µm was selected to assess the effect of bone shrinkage during the embedding process on BIC; 30 µm were selected assuming a shrinkage to the extent of the average size of an osteoblast. The enlargement by 1 mm was performed to reduce the effect that the screw thread shape may have on bone formation. Therefore, 1 mm was selected, which is approximately double the size of the threads. Enlarging the non-degraded screws and quantifying the voxel numbers was performed in Fiji. The enlargement was based on applying a distance map to the non-degraded screw and selecting all voxels with a distance of ≤ 1 mm from the original screw surface.

Finally, BV/TV was determined as [25, 26]:

(3) $$\frac{BV}{TV}=\frac{\#\;\mathrm{voxels}\;\mathrm{of}\;\mathrm{bone}\;\mathrm{in}\;\mathrm{ROI}\;}{\#\;\mathrm{voxels}\;\mathrm{of}\;\mathrm{bone}+\mathrm{background}\;\mathrm{in}\;\mathrm{ROI}}$$

### Histological examination

Tissue samples containing the screws were first cut into blocks of ∼30 mm thickness using an EXAKT precision saw (EXAKT Apparatebau, Norderstedt, Germany), followed by tissue fixation in 4% neutral-buffered formaldehyde. After fixation, the specimens were dehydrated in ascending grades of ethanol. Samples were then embedded into resin (methylmethacrylate, nonylphenyl-polyethyleneglycol acetate, benzoyl peroxide, Sigma-Aldrich, Merck KGaA, Darmstadt, Germany). Undecalcified thin-ground sections along the longitudinal axis of the screws in the frontal plane of the tibia shaft were produced according to the method of Donath [27], using EXAKT cutting and grinding equipment (EXAKT Apparatebau, Norderstedt, Germany). For Giemsa staining, polished thin ground sections were etched for 2 min in 0.1% formic acid, rinsed in distilled water, submersed in 20% methanol for 15 min and rinsed again in distilled water. Staining took place for 30 min in a 20% Giemsa solution (Merck KGaA, Darmstadt, Germany) in distilled water at 60°C. Two final rinsing steps were performed, consisting of rinsing with acidic solution (three drops acidic acid/100 ml distilled water) followed by distilled water. Samples were digitized with an Olympus BX53 microscope automatic system and the software OLYMPUS cellSens Dimension 3.1 (Olympus, Tokyo, Japan).

## Results

### Qualitative analysis of implant degradation and bone structure in different implantation sites

#### Descriptive analysis of in vivo cCT

**Figure 3.**
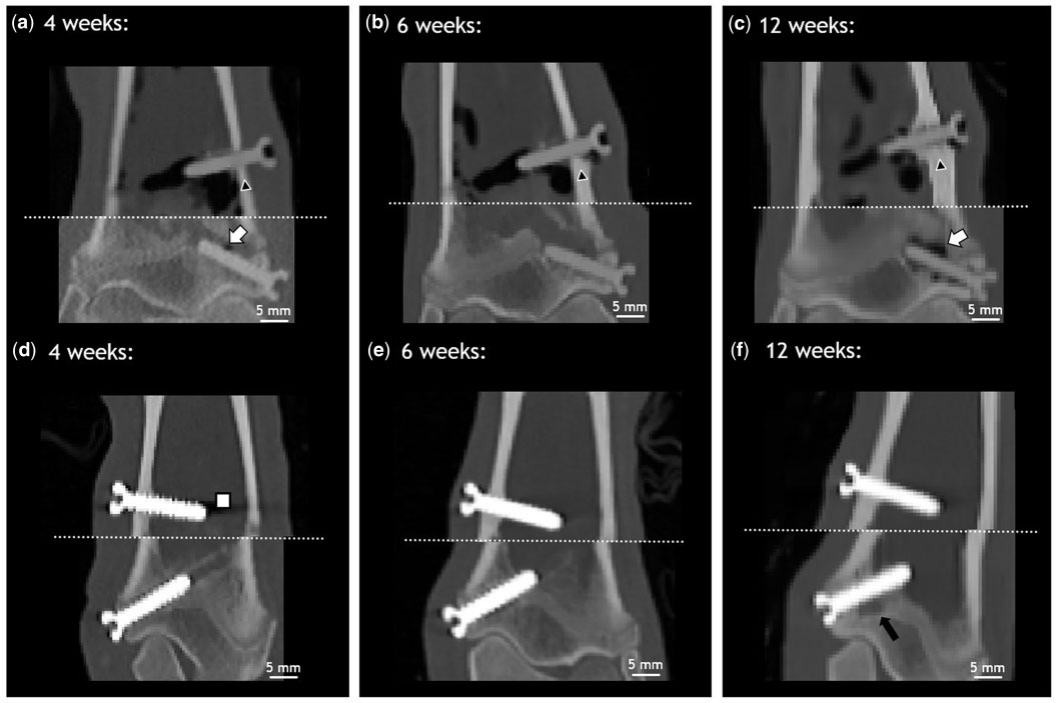
*In vivo* cCT images of distal tibiae of one animal. ZX00 (**a–c**) and Ti (**d–f**) screws 4, 6 and 12 weeks after implantation are shown. White arrows indicate gas formation around the epiphyseal screw (a and c). Cortical thickness increases over time (a–c; black triangle). Some scanning artifacts (white square) were detected around the Ti-screws, mainly at the screw tip 4 weeks after implantation (d). Slight change in trabecular bone structure was detected below Ti-screw after 12 weeks (f; black arrow).

*In vivo* cCTs were performed 4, 6 and 12 weeks after surgery, allowing tracking of the degradation behavior within the same animal over time. Figure 3 shows only cCT images at these time points for the animal that was euthanized after 12 weeks. cCT images of the remaining animals are listed in the supplementary data (Supplementary Fig. S2). Images of the metaphyseal and epiphyseal screws of the same animal were stitched together. The stitching line is depicted as a white dotted line. Gas formation was observable already 4 weeks after implantation, mostly apparent around the metaphyseal screw (Fig. 3a). However, a small radiolucent area was present around the epiphyseal screw after 4 weeks (Fig. 3a; white arrow), which was more pronounced after 12 weeks (Fig. 3c; white arrow). Small gas pockets were observable in the soft tissue around ZX00-screw heads for all animals and all time points. We observed an increase in cortical thickness in the vicinity of the metaphyseal ZX00-screw over time, which was more pronounced distal of the screw (Fig. 3a–c; black triangle). The increase in cortical thickness around Ti-screws was less distinct. Slight changes in trabecular bone structure were found below the Ti-screw 12 weeks after implantation (Fig. 3f; black arrow). Scanning artifacts were solely found around Ti-implants, mainly around the screw tips, but also around the screw head (Fig. 3d–f).

#### Descriptive analysis of ex vivo µCT

**Figure 4.**
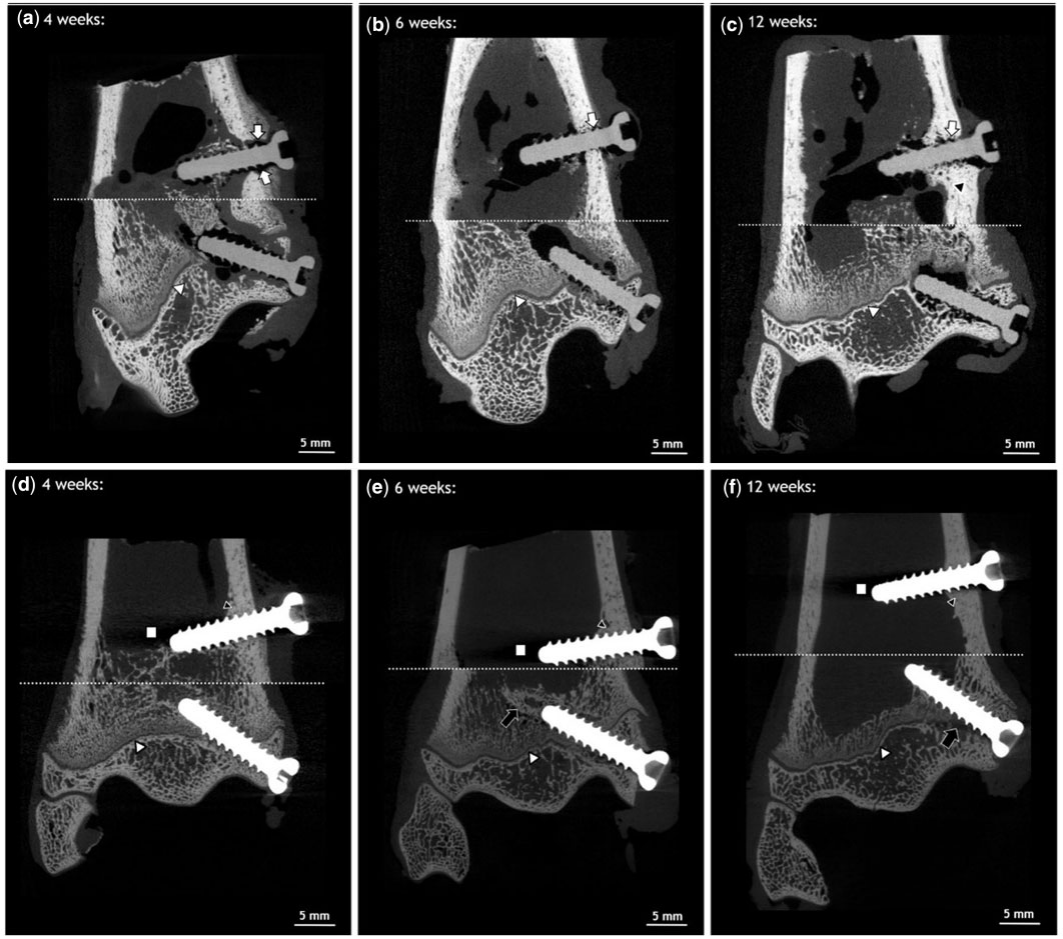
*Ex vivo* µCT images of distal metaphysis and epiphysis. ZX00 (**a–c**) and Ti (**d–f**) screws 4, 6 and 12 weeks after implantation. Images of metaphyseal and epiphyseal screws of the same animals were stitched together. The stitching line is marked with a white dotted line. Increased cortical thickness was detected around ZX00-screw after 12 weeks (black triangle). Small gas pockets were found in the cortical bone around the ZX00-screw after 4, 6 and 12 weeks (white arrows). Slight alterations in trabecular bone structure around Ti-screws were detected 6 and 12 weeks after implantation (e and f; black arrows). Scanning artifacts were found only around Ti-implants (d–f; white squares). The physis was detected in all animals (a–f; white triangle), interrupted by the implant.

*Ex vivo* µCT scans were performed on all the samples after removal of the proximal part and the shaft of the tibiae. Four weeks after implantation, gas evolution was mainly visible around the screw tip, but also some smaller gas pockets were detected below the screw head within the cortical bone area (Fig. 4a; white arrow). In both meta- and epiphysis, a gap was visible between implant and bone structure in the cortical and trabecular bone areas at 4 weeks (Fig. 4a). The drillhole from bicortical drilling was still present in the contralateral cortex. After 6 weeks, more bone structure was visible in both implantation sites directly in the vicinity of the screws (Fig. 4b). The bone defect in the area of the drilling hole in the contralateral cortex was still detectible, but already closed. Gas pockets were detected around both, meta- and epiphyseal screws after 6 weeks, and appeared to be more distinct around the screw tips. After 12 weeks, gas formation was more pronounced in the metaphyseal area when compared with the other screw and time points (Fig. 4c). The crests of the screw threads of both meta- and epiphyseal screws appeared smoother at this time point. Furthermore, the cortical bone surrounding the ZX00-screw was thicker when compared with the rest of the cortex, but also when compared with the earlier time points (Fig. 4c; black triangle). This correlates with the findings in the cCT. The physis was visible in all three animals (Fig. 4a–f; white triangle) and appeared to be interrupted by the screws.

The bone structure around Ti-implants appeared homogeneous, except for a minor thickening of the cortex in the vicinity of the metaphyseal screws (Fig. 4d–f; black triangle). The corticalis around the Ti-screw 12 weeks after implantation was thinner when compared with the corticalis around the ZX00-screw at the same time point. After 6 and 12 weeks, slight alterations in trabecular bone areas were detected around the Ti-screws (Fig. 4e and f; black arrow). Scanning artifacts were visible at all time points in the area of the Ti-screw tips (Fig. 4d–f; white square). Physis were also visible in all three animals and were interrupted by the screw, similar to the surrounding of ZX00-screws (Fig. 4d–f; white triangle).

### Zx00 degradation performance in different bone regions, 4, 6 and 12 weeks after implantation

#### Surface area, implant volume and DR in different bone regions evaluated by μCT

**Figure 5.**
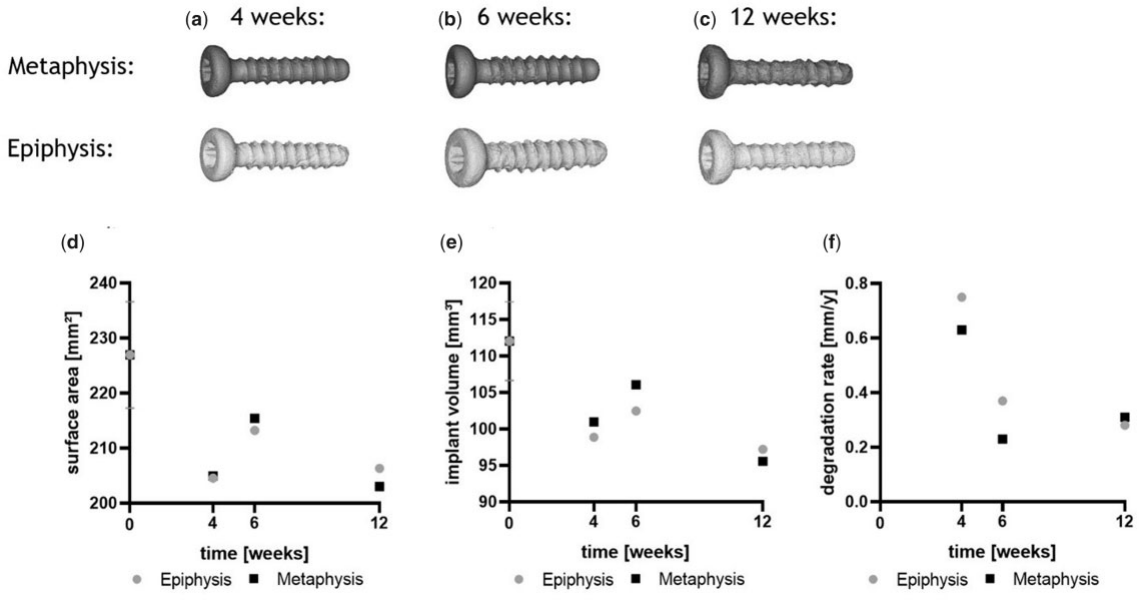
3D-models and calculated implant characteristics of ZX00-screws over time; 3D-models segmented from µCT data are shown in (**a–c**). Surface area (**d**), implant volume (**e**) and resulting degradation rate (**f**) of ZX00-screws in epiphysis and metaphysis 4, 6 and 12 weeks after implantation were calculated from µCT data.

Segmented µCT data were used to create 3D-surface-models of the screws and to calculate implant and gas volume over time. First alterations at the implant surface occurred mainly in the screw thread area (Fig. 5a). The thread of the epiphyseal screw revealed a rougher surface when compared with the metaphyseal screw 4 and 6 weeks after implantation. In contrast, the surface of the metaphyseal screw appeared rougher when compared with the epiphyseal screw, 12 weeks after implantation. The screw heads appeared smoother when compared with the thread area at all time points and in both implantation sites.

Surface areas of meta- and epiphyseal screws were comparable 4 weeks after degradation (numbers are given in Supplementary Table S1), whereas the decrease in surface area was less pronounced in both implantation sites 6 weeks after implantation. After 12 weeks, the surface areas were comparable to the 4-week time point in meta- and epiphysis.

Accordingly, only slight differences were found between the implant volumes in the epi- and metaphysis 4 weeks after implantation, respectively (Fig. 5e). After 6 weeks, VL was slightly more pronounced in the epiphyseal screw when compared with the metaphyseal screw (Fig. 5e). The highest VL was found after 12 weeks in both implantation sites (numbers are given in Supplementary Table S2).

The DR was calculated for both implantation sites and all time points, using equation (1). The highest DR was observed for the screws after 4 weeks in both implantation sites, which decreased at 6 weeks post-implantation (Fig. 5f). However, the DR further decreased in epiphysis, whereas we observed a slight increase in the metaphysis, 12 weeks after implantation. The lowest DR was found in the metaphysis at the 6-weeks’ time point (numbers are given in Supplementary Table S3).

#### Gas evolution in different bone regions evaluated by µCT

**Figure 6.**
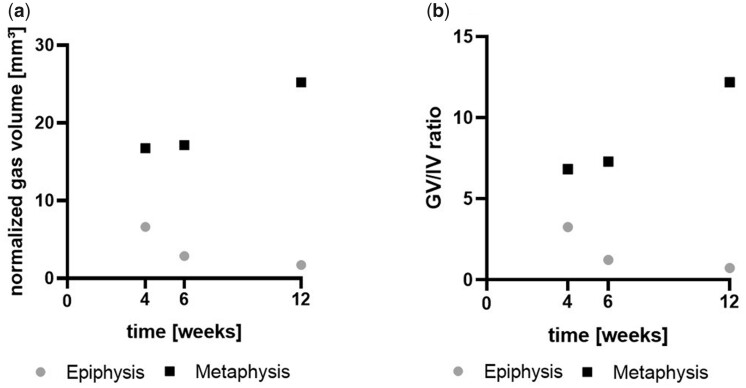
Gas volume and GV/IV evaluated from µCT data. (**a**) Shows the gas volume of the screws within the normalized ROIs and (**b**) the ratio of gas volume to implant volume within the normalized ROIs.

As epiphyseal and metaphyseal screws were implanted close to each other, gas pockets from the screws sometimes fused, which disabled distinguishing between them. Thus, we only evaluated gas volume evolution and GV/IV within predefined ROIs around the screws (numbers are given in Supplementary Tables S5 and S6). The gas evolution was more pronounced in the metaphyseal area when compared with the epiphyseal area at all time points (Fig. 6a). In the metaphysis, normalized gas volume increased over the implantation time. However, a decreasing trend was detected in the epiphysis. The largest difference in gas volume between epiphyseal and metaphyseal screw was detected 12 weeks after implantation, when the normalized gas volume was almost 15 times higher in the metaphysis when compared with the epiphysis. The same pattern was shown for the GV/IV ratio (Fig. 6b).

### Zx00 degradation performance in different bone types, 4, 6 and 12 weeks after implantation

#### VL and DR in different bone types evaluated by SRμCT

**Figure 7.**
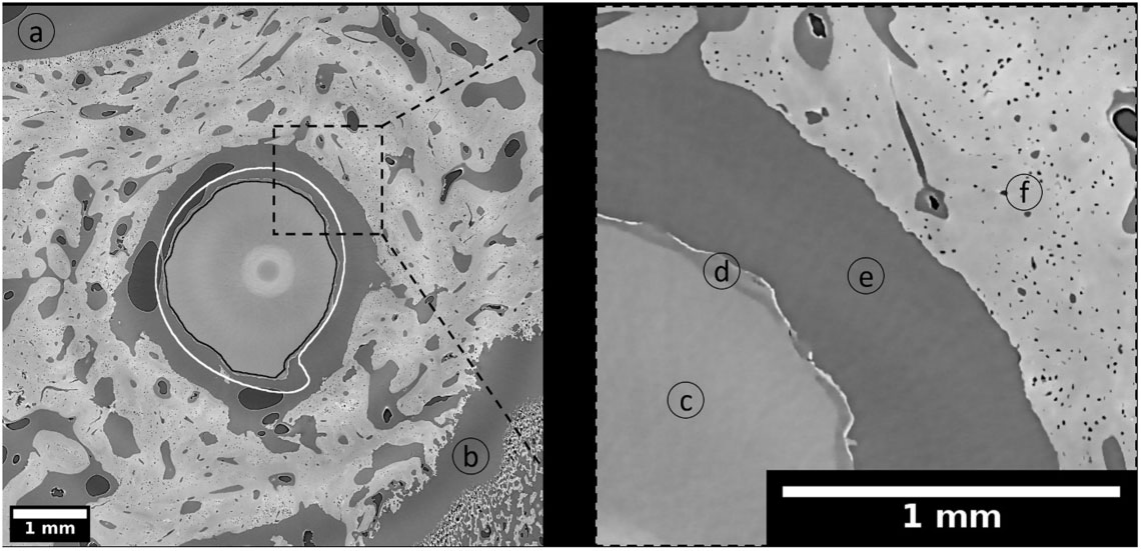
Cross section of ZX00-screw implanted in distal epiphysis (dpROI) after 12 weeks of healing. The black line is an outline of the segmented residual metal and the white line is an outline of the non-degraded, registered and resampled screw. Highlighted details: background (**a**) and (**e**), epiphyseal line (**b**), residual metal (**c**), degradation layer (**d**), trabecular bone with blood vessels and osteocyte lacunae (**f**). Effective pixel size obtained with the use of SRµCT was 3.18 µm. The bright circle in the middle of the image is an artifact from stitching the images.

The screws implanted into the epiphysis and metaphysis were surrounded by different types of bone, which were divided into four sub-ROIs for analysis, as defined in Section ‘Micro computed tomography’. In order to perform the calculations, we distinguished between residual material (non-corroded alloy), degradation layer (corrosion products attached to the residual material), bone (mineralized tissue) and background (all remaining features not assigned to previously mentioned labels). Exemplary SRµCT images depicting selected cross sections of explants with ZX00-screws for dpROI, ppROI, cROI and iROI after 4, 6 and 12 weeks of healing are presented in the supplementary data (Supplementary Fig. S3). The overview images give information about the bone morphology around degraded ZX00-implants. Figure 7 depicts an example of the degraded ZX00-alloy at the 12-week time point, in comparison to the non-degraded, registered and resampled screw whose outline is marked in white. The black outline highlights the segmented residual metal. The high-resolution SRµCT scans revealed a thin and locally cracked degradation layer. Similar to the µCT data, the surface of the degraded metal was losing its smoothness and the threads their sharpness. In the magnified section of Fig. 7, the corroded metal and degradation layer are visible, as well as the surrounding trabecular bone with blood vessel and osteocyte lacunae.

**Figure 8.**
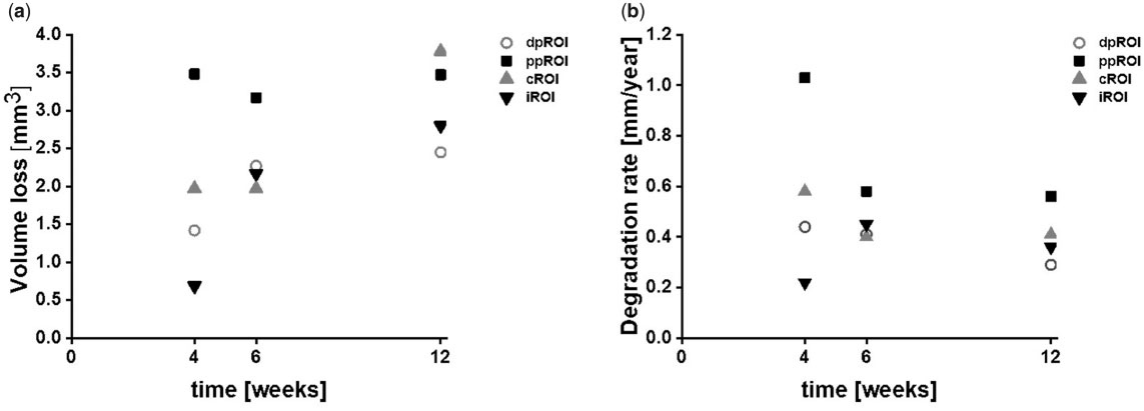
VL and DR of ZX00-implants over time. (**a**) Volume loss and (**b**) degradation rate of each ROI depending on the healing time from SRµCT images. dpROI: trabecular bone distal to physis; ppROI: trabecular bone proximal to physis; cROI: cortical bone; iROI: intermedullary cavity.

Figure 8 displays the corresponding quantitative measurements of VL and DR. For dpROI, a slight increase of VL was observed, which resulted in a linear trend for the DR (Fig. 8a and b, numbers are given in Supplementary Tables S3 and S4). Thus, the smallest DR value was found after 12 weeks of degradation. A different trend was observed for ppROI—high VL occurred for each time point, revealing the fastest DRs after 4 weeks. After 12 weeks, minimum and maximum DR were both found in the trabecular bone area, namely in dpROI and ppROI, respectively. Considering the results from metaphyseal implantation site, the highest VL was observed for cROI after 12 weeks. The biggest difference in VL between the time points was found in iROI. DR for cROI and iROI were comparable after 6 and 12 weeks of implantation.

#### Gas evaluation in different bone types evaluated by μCT

**Figure 9.**
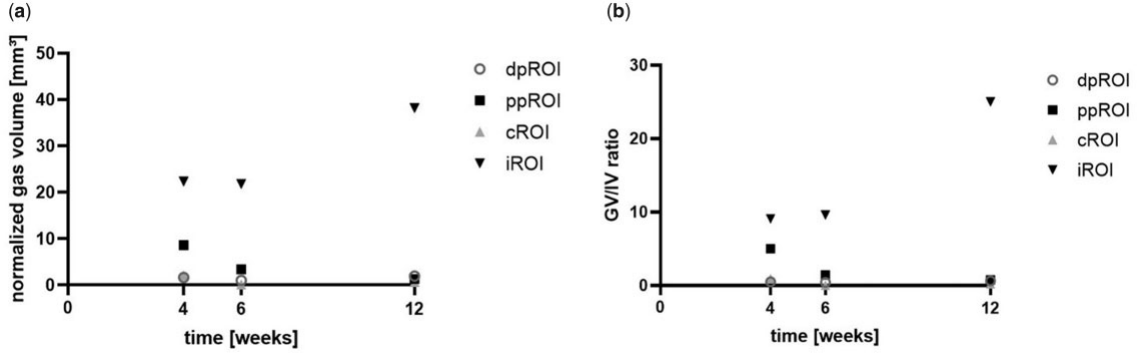
Gas volume and GV/IV 4, 6 and 12 weeks after implantation. Gas volume (**a**) and GV/IV ratio (**b**) were calculated for each normalized ROI from µCT data.

Gas evolution was calculated for each sub-ROI, using µCT data (numbers are given in Supplementary Table S5). The highest gas volume and GV/IV was found in iROI at all time points (Fig. 9a and b). After 4 weeks, GV/IV in iROI almost doubled GV/IV from ppROI, whereas being more than 10 times higher when compared with the ratios in dpROI and cROI. After 6 weeks, the difference was even more pronounced. Twelve weeks after implantation, GV/IV of iROI was more than 30 times higher when compared with the other ROIs. The lowest normalized gas amount was found in the cortical bone area at all time points.

#### BIC and BV/TV in different bone types evaluated by SRμCT

**Figure 10.**
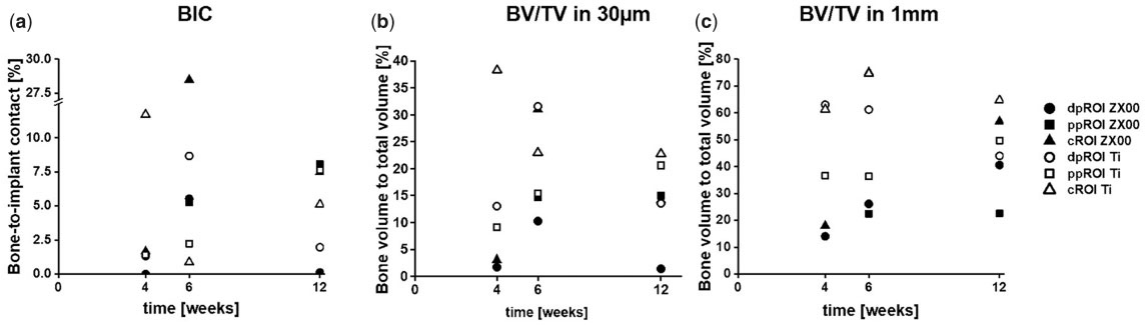
BIC and BV/TV of ZX00 and Ti-implants over time. (**a**) BIC and (**b**) BV/TV in a 30 µm distance from non-degraded screw and (**c**) BV/TV in a 1-mm distance from non-degraded screw for ZX00 and Ti-screws in the selected ROIs after 4, 6 and 12 weeks of healing times. dpROI: trabecular bone distal to physis, ppROI: trabecular bone proximal to physis; cROI: cortical bone.

The osseointegration was investigated with high precision in 3D for the selected ROIs using SRµCT to quantify BIC and BV/TV (Fig. 10) in the different bone types. For iROI, BIC and BV/TV parameters were not calculated, as there was no mineralized bone detected in the intramedullary cavity. The overview of bone formation around the ZX00- and Ti-implants is shown in the Supplementary Figs S3 and S4 and the visualization of chosen area for quantifications of BV/TV is shown in the Supplementary Fig. S5.

For the ZX00-screw in dpROI and ppROI, no BIC was calculated 4 weeks after implantation, as no bone contact was found. Furthermore, a gap between the screw and the tissue was detected in ppROI, 4 weeks after implantation. The tissue boundary possesses the shape of the screw thread, indicating that the screw moved after implantation (Supplementary Fig. S3). Most of the measurements revealed a BIC of < 10% irrespective of the ROI and material. The highest BIC was found for the ZX00-sample in cROI after 6 weeks of healing, followed by the BIC of the Ti-sample in cROI after 4 weeks of healing. After 12 weeks, the highest BIC was found in ppROI around the ZX00-screw, closely followed by BIC around Ti-screw in ppROI and BIC around ZX00-screw in cROI. Lowest BIC was found in dpROI around ZX00-screws after 4 and 12 weeks, followed by the BIC in cROI around the Ti-screw 6 weeks after implantation (numbers are given in Supplementary Table S7).

BV/TV close to the implant was assessed for comparison to BIC, due to potential shrinkage of the bone during embedding. Figure 8b shows higher bone volume surrounding Ti-implants for most of the ROIs—the highest percentage was noted for cROI after 4-week implantation time. The same trend is visible for BV/TV in a larger volume surrounding the implant (Fig. 10c). An increasing BV/TV around ZX00-samples was observed for dpROI and cROI. A stable bone volume was found in ppROI of ZX00-screws 6 and 12 weeks after implantation. In dpROI, the ZX00- and the Ti-samples showed diverging values for the early time points but arrived at a similar level after 12 weeks of healing. For the cROI of Ti, a slight decrease of BV/TV values over the implantation time were observed close to the implant, as well as for dpROI in a larger volume surrounding the implant (numbers are given in Supplementary Tables S8 and S9).

#### Descriptive histological evaluation of implants in different bone types

**Figure 11.**
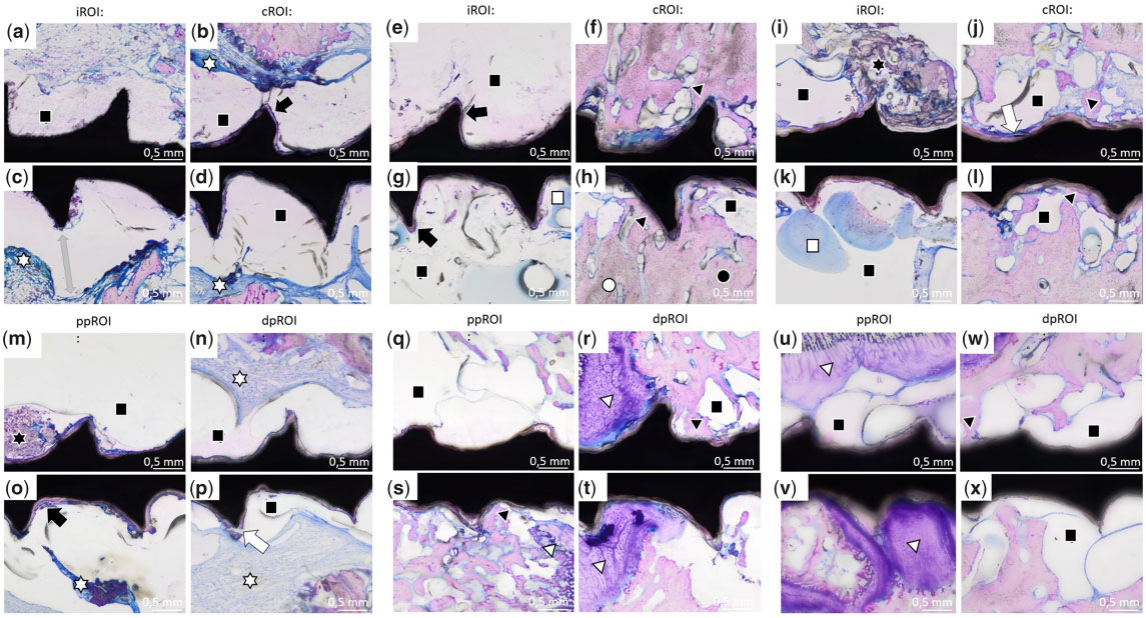
Histology images of ZX00-screws. Sections from iROI and cROI around ZX00-screws 4 weeks (**a–d**), 6 weeks (**e–h**) and 12 weeks (**i–l**) after implantation, as well as of ppROI and dpROI 4 weeks (**m–p**), 6 weeks (**q–t**) and 12 weeks (**u–x**) after implantation. Some void areas (black squares) were detected around the screws in all different ROIs. A gap between the ZX00-screw and the cells was found 4 weeks after implantation (c; gray arrow). The corrosion layer was visible in different areas around ZX00-screws (b, e, g and o; black arrow). Small cracks were sometimes detected in the corrosion layer (j and p; white arrow). Direct BIC (black triangle) can be seen in several areas of cROI, as well as ppROI and dpROI. In (h), it can be distinguished between old bone (white circle) and newly formed bone (black circle). In few areas, the histological slice detached from the surface, causing an artifact (g and k; white square). Inflammatory cells were found around ZX00-screws 4 weeks after implantation (b, c, d, n, o and p; white star).

**Figure 12.**
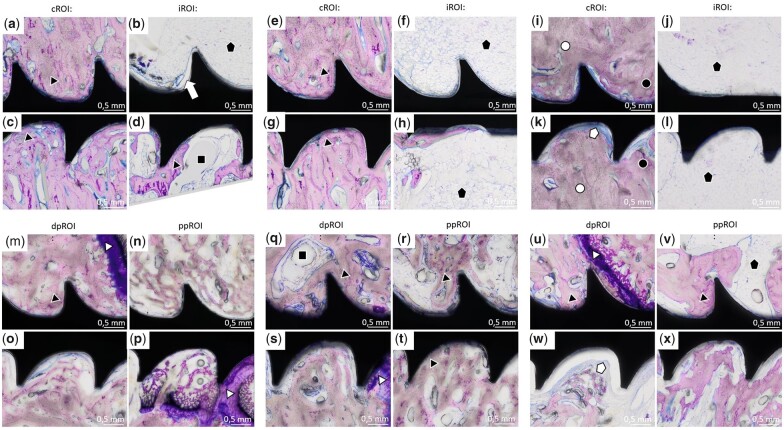
Histology images of Ti-screws. Sections of iROI and cROI around Ti-screws 4 weeks (**a–d**), 6 weeks (**e–h**) and 12 weeks (**i–l**) after implantation, as well as dpROI and ppROI 4 weeks (**m–p**), 6 weeks (**q–t**) and 12 weeks (**u–x**) after implantation. Some void areas (black squares) were detected around the screws in all different ROIs. Direct BIC (black triangle) can be seen in all different ROIs at all time points. In iROI and cROI 12 weeks after implantation, it can be distinguished between old bone (white circle) and newly formed bone (black circle) (**i and k**). Fat cells (black pentagram) were mostly found in iROI (b, f, j, h and l), but were also detected in ppROI (v). In some areas, a small layer of connective tissue formed between bone and implant (k and w; white pentagram).

Histological analysis was performed of all samples. Overall images of the whole screw with surrounding tissue are listed in the Supplementary Data (Supplementary Fig. S6). Magnifications shown in Figs 11 and 12 focus on the four sub-ROIs previously defined. Four weeks after implantation, the histological analysis of ZX00-screws revealed connective tissue with some inflammatory cells (mostly lymphocytes) around both metaphyseal (Fig. 11a–d; white star) and epiphyseal (Fig. 11n–p; white star) screws. In addition, no direct BIC was found in accordance with SRµCT images. In order to ensure that inflammatory reactions do not occur regularly around ZX00-implants at this postoperative stage, we investigated one additional ZX00-screw (40 × 3.5 mm) from the same time point but from the proximal epiphysis of a different animal (Supplementary Fig. S7). Histological examination was performed in the same manner. No inflammatory cells were found around this screw. Void areas without any cell content were detected around the ZX00-screws in all different ROIs (Fig. 11a–x). Four weeks after implantation, a ∼1.5 mm wide gap between cells and implant surface was detected (Fig. 11, gray arrow). A corrosion layer was visible in several areas around the ZX00-screws (Fig. 11b, e, g and o; black arrow). In accordance with the SRµCT data, small cracks in the corrosion layer were detected (Fig. 11j and p; white arrow). After 6 and 12 weeks, BIC was visible at meta- and epiphyseal screws (Fig. 11f, h, j, s, r and v; black triangle). Six weeks after surgery, areas containing old bone, as well as newly formed bone were detected in the cortex around the ZX00-screws (Fig. 11h). Cortical bone around ZX00-screws contained several void areas after both 6 weeks (Fig. 11f and h; black square) and 12 weeks (Fig. 11j and l; black square). In some areas, debris was found around the screw threads (Fig. 11i and m; black star). Some histological slices detached slightly from the sample holder leading to artifacts, which stained blue (Fig. 11g and k; white square). The chondrocytes forming the physis were found around ZX00-screws 6 and 12 weeks after implantation (Fig. 11r, t, u and w; white triangle). In some cases, chondrocytes were in direct contact with the implant surface (Fig. 11r, t and w). A completely intact physis was observed around the ZX00-screw 12 weeks after implantation.

Histological analysis of Ti-screws revealed direct BIC in epi- and metaphyseal screws at all time points (Fig. 12a–x), which confirms the findings of the SRµCT data. In iROI, fat cells were found in the vicinity of the screws (Fig. 12b, f, h, j, l, v; black pentagram), which were unfrequently detected in iROI around ZX00-screws. Additionally, around the Ti-screws, some void areas without any cell content were found (Fig. 12d and q; black square). Furthermore, a small layer of connective tissue without inflammatory cells formed between the bone tissue and the implant (Fig. 12k and w; white pentagram). The physis around Ti-implants was found to be thinner when compared with the physis around ZX00-implants, especially 12 weeks after implantation (Fig. 12u). In the cortical bone area around the Ti-screws, older bone (white circle), as well as newly formed bone (black circle) was found (Fig. 12i and k). After 6 and 12 weeks, the cortex around Ti-screws appeared to be thinner, but denser when compared with cortex around ZX00 at same time points, which confirms the findings of the µCT data. Further on, 12 weeks after implantation, mineralized bone tissue was observed in the physis gap next to the Ti-screw, forming a bone bridge (Supplementary Fig. S6l; black triangle).

## Discussion

In this study, we investigated the differences in degradation behavior of ZX00-screws in relation to their implantation site thereby focusing on the impact of different bone regions and bone types (epiphysis, physis, metaphysis and intramedullary cavity). Implant degradation evaluated by VL and DR was accelerated in the epiphysis when compared with the metaphysis, 4 and 6 weeks after implantation. As shown in the histological analysis, ZX00-implants within iROI were often surrounded by void areas, without any cell content. cCT and µCT data revealed gas formation around ZX00-screws in iROI at all time points. Thus, we suggest the formation of the present voids around ZX00-screws due to gas evolution. Consequently, an inhibition in electrochemical and cellular interactions within these regions occurs, resulting in a decreased DR when compared with the DR in the epiphyseal area. Furthermore, the main part of the epiphyseal screws lay within trabecular bone structure, which is infused with a large content of blood vessels [12, 28]. Thus, increased perfusion around the implant enables promoted ion exchange between the implant material and the surrounding tissue, facilitating the degradation. Thread crests of the epiphyseal screws were smoother when compared with the metaphyseal screws, indicating advanced implant corrosion within the epiphysis. Similar findings were published by Kraus *et al*. [29]. They reported increased degradation in the epiphysis when compared with the metaphysis, after investigating the *in vivo* degradation of Mg alloys in distal rat femora. Holweg *et al*. described initially enhanced implant degradation in the epiphyseal area of sheep tibiae, when compared with the diaphyseal area [11]. In this study, we implanted into the metaphysis rather than into the diaphysis. However, the screw tip reached into the intramedullary cavity, similar to diaphyseally implanted screws. Therefore, the screws were exposed to similar tissue types, allowing a comparison between the studies. The DRs calculated for both implantation sites accounted 0.23–0.75 mm/year. This lies within the range of DRs of different Mg alloys found in the literature [30, 31]. Chaya *et al*. stated that they achieved fracture healing with DRs of 0.4 mm/year, using pure Mg implants in a rabbit model [32] indicating suitability for bone repair applications. Furthermore, we calculated the DRs from the values found in the work of Holweg *et al*. [8] using the equation defined under Section ‘Micro computed tomography’. According to the numbers found in their work, the DRs ranged from 0.21 to 0.83 mm/year, which was sufficient for fracture healing in a sheep model, using ZX00 implants. Here, the fastest DR for the ZX00-screws was found 4 weeks after implantation in both, epiphysis and metaphysis. However, previous *in vivo* studies with ZX00-alloy showed that the DR usually decreases over time [7, 11], which correlates with our findings. As BIC increases over time, ion exchange decreases, resulting in a decelerated DR. Thus, it is difficult to estimate the exact time needed for the implants to be fully resorbed. To elaborate the exact DR value, a long-term study is needed, which also takes the degradation inhomogeneity within the different bone regions into account.

The evaluations of the implant volumes over time revealed only small differences between the implantation sites. The biggest difference was found 6 weeks after implantation, with a difference of 3.5%. We consider this difference in implant volume between the implantation sites to be below clinical relevance.

Investigation of gas evaluation revealed distinct differences between the implantation sites and bone types. Gas evolution was more pronounced around the metaphyseal screws when compared with the epiphyseal screws at all time points, with the highest gas volumes found in iROI. Interestingly, the gas volume in iROI did not correlate with the DR in the same area, which was actually low. We suggest that this is due to the screw positioning in the bone. The heads of the implant protrude from the bone into the soft tissue. Thus, hydrogen gas generated in the cortex and trabecular bone can easily divert into the soft tissue. We found small gas pockets in the soft tissue around the screw heads in the cCT, which supports this theory. In addition, forming gas voids encounter less resistance within the soft tissue and bone marrow cells when compared with the cortical bone. Thus, it is likely, that a considerable amount of hydrogen gas proceeds into the soft tissue or into the medulla, where gas pockets are trapped. Several studies reported considerable gas formation within the intramedullary cavity. Rössig *et al*. inserted LAE443 implants into the tibiae of sheep [33]. They reported highest gas accumulation in the medulla and in the soft tissue. Kraus *et al*. reported excessive gas formation within the medulla after implantation of fast degrading ZX50 pins into rat femora [34]. Though, less gas formation was found around slow degrading WZ21 pins in the same study. Grün *et al*. described gas accumulations mainly in the soft tissue, but also within diaphyseal trabecular bone structures around ZX00 pins implanted into rat femora [7]. Furthermore, they found pronounced gas formation within the bone marrow around ZX00-implants in sheep tibiae. Another study with W4 implants depicted excessive gas formation within the epiphysis, which even led to implant loosening [35]. However, we found no studies, which performed a comparison between the gas formation in epiphysis and metaphysis. Holweg *et al*. reported that no significant difference in gas formation between epi/-metaphyseal and diaphyseal screws was found, using the same alloying system in a sheep model as we did [11]. Yet, they did not differentiate between epiphysis and metaphysis.

In order to suffice as implant material for bone repair applications, the DR must be low enough for the bone to allow gradual replacement of the degraded regions by tissue, to guarantee stability. Additionally, direct contact between the implant and the tissue is necessary, in order to take over some of the mechanical load occurring in the bone until fracture consolidation. The implant is stabilized both by calcified bone, as well as cartilage tissue, which typically form during callus formation [36]. Here, direct BIC was found around both ZX00 and Ti-screws. For both materials, the minimum and maximum values changed between the bone types over time. Thus, we did not find a clear trend for the BIC between the implantation sites and bone types for either material. Yet, the fact that we found comparable BIC for Mg and Ti suggests ability of the bone to form sufficient contact for load distribution during the healing process, which suggests suitability for bone repair applications. Schaller *et al*. investigated the *in vivo* degradation of coated and non-coated WE43 screws in a minipig model [37]. They reported significantly lower BIC and BV/TV for Mg screws when compared with Ti-screws 12 and 24 weeks after implantation. BIC of Mg screws was comparable to Ti when they were coated, with a delayed degradation. Another study in the frontal bone of minipigs demonstrated that BIC of the WE43 implants was again significantly lower when compared with the Ti-screws 12 and 24 weeks after implantation [38]. By contrast, Krüger *et al*. found similar BIC of Mg-xGd implants to Ti-implants in a rat study [13]. Castellani *et al*. reached a higher BIC when compared with Ti-implants after 4, 12 and 24 weeks, using a Mg-Y-Nd-HRE alloy in a rat model [39]. Rare earth elements (REE) are known to decelerate the DR [40, 41]. However, previous studies show that REEs can accumulate in the organs [33, 42, 43]. To date, literature concerning the impact of REE accumulations on the health in the long term is sparse. Nevertheless, several studies state the negative influence of REE not only on organs, but also on cell lines and physiological processes [40, 44, 45]. Therefore, we decided to use REE-free implants for this study.

In case of the BV/TV, we found higher values around Ti-samples. On the other hand, we found an increased cortical thickness around the ZX00-screws when compared with the Ti-screws in cCT and µCT data after 6 and 12 weeks. For the screw stability, the BIC over the whole screw needs to be considered. Therefore, the increased cortical thickness can outweigh the slightly decreased BV/TV from the observed ROI. This is supported by the findings of Holweg *et al*. [8] and Herber *et al*. [9]. They showed highly satisfactory results in two clinical studies using ZX00-screws for fracture treatment. Furthermore, complete fracture consolidation was shown 12 weeks post-surgery after stabilization with ZX00-screws in a sheep model [11]. To obtain further information on the biomechanical stability, biomechanical pullout tests are warranted, in order to correlate the BIC with the force needed, to extract the screws.

As shown in the histological analysis, inflammatory cells were found around ZX00-screws 4 weeks after implantation, indicating early infection. No swab was taken from the implantation site, as we found the signs of inflammation only after performing histology. Therefore, the potential presence of bacteria remains unknown. Yet, no maturations were detected from the implantation site. Clinical studies reveal an overall infection rate of 5% for internal fixation devices [46]. Despite thorough wound disinfection after the surgery, sheep are prone to infections, as the wounds can hardly be kept clean after the surgery, which is different for humans. Nevertheless, as postoperative wound infections cannot always be prevented, appropriate DR under infected conditions needs to be guaranteed as well. It is known that infections can lead to a drop in pH in the tissue, due to aerobic consumption [47, 48]. We suggest that the high DR revealed for the ZX00-screws 4 weeks after implantation occurred due to a drop in pH during inflammatory reaction. Besides accelerating the DR, acidosis also inhibits osteoblastic functions and accelerates osteoclastic activity *in vitro* [49], thus decreasing bone ingrowth, which could be an explanation for the poor BIC at this time point. However, it must be considered that in case of a strong inflammatory reaction or an infection, a similar effect is seen also around other materials, such as Ti or PEEK [50, 51] and is therefore rather caused by this reaction than the material itself. The poor BIC could impede distribution of mechanical load within the bone at this time point. Nevertheless, only movement stability and not load stability is necessary in case of this surgery technique, reducing the need for a high contact. Furthermore, to ensure that infections are not regularly associated with Mg implantation, one additional ZX00-screw from a different animal at the same time point was investigated. The screw was implanted into the proximal, instead of the distal epiphysis of a growing sheep. As this screw only served as a control concerning occurrence of inflammatory cells within the surrounding tissue at depicted time point, the difference in implantation site was considered to be negligible. No inflammatory cells were found around this screw (Supplementary Fig. S7). Furthermore, ZX00-implants were already investigated in a sheep study by Holweg *et al*. [8] and in a rat and sheep study by Grün *et al*. [7]. Neither of those studies reported findings of inflammatory cells around ZX00-implants in the histological examinations. Thus, we suggest that inflammatory reaction in our study depicted around ZX00-screws 4 weeks after implantation represents an outlier.

A bone bridge was detected in the physis around the Ti-screw 12 weeks after implantation (Supplementary Fig. S6). As the physis is the area where longitudinal growth of the long bones takes place, a bone bridge often leads to growth disturbances in this specific area. The formation of the bone bridge is a known potential complication of physeal injuries and non-resorbable transphyseal implants [52–54]. Importantly, there was no bone bridge observable around epiphyseal ZX00-screws, suggesting that growth mechanisms remain unaffected. In contrast to the Ti-screws, a thicker physis was detected around ZX00-screws. Nevertheless, further investigations about the influence of Mg on chondrocytes and thus, on the physis, of ZX00-implants are needed.

This study is mainly limited by its low sample number, and therefore only serves as a road map for future studies. No statistical evaluations were carried out. Thus, additional studies with higher animal numbers are needed, in order to confirm current conclusions. A further limitation is the performed method for the investigation of gas evolution around the implant. CT data only reveal a momentary situation. The overall gas formation over time thus remains unknown. Furthermore, the calculations of BIC and BV/TV were performed only for small ROIs. However, we showed that the cortical bone around ZX00-screws is less dense, but thicker, when compared with the cortex around Ti-implants. Therefore, we suggest to calculate BIC and BV/TV over the whole screw. In addition, SRµCT and histological samples possessed small cracks in the corrosion layer. It is known that during embedding processes, a shrinkage of the polymerized block containing the tissue sample occurs [55]. As the metal implant retains its size, this can lead to a detachment of the corrosion layer, as well as the tissue from the implant surface, which can lead to misinterpretations of the results. Yet, embedding of the samples is necessary in order to perform histology, and to the best of our knowledge, there is no method, which could prevent tissue shrinkage.

## Conclusion

In conclusion, we suggest the suitability of ZX00-screws for implantation into the distal meta- and epiphysis. The ZX00-implants revealed satisfactory DRs and gas formation in all investigated bone areas, making it a suitable material for orthopedic implants. In case of the BIC, no clear trend was found between the implantation sites and materials. Higher BV/TV was found around Ti-implants in pre-defined ROIs. However, we found an increased cortical thickness around ZX00-screws when compared with Ti-screws. We suggest performing of biomechanical pullout tests with ZX00-implants, in order to elaborate the attachment of the screws to the bone.

In addition, the absence of a bone bridge formation in the physis around ZX00-screws could make it a potential implant material for the treatment of pediatric fractures, occurring around the physis. Thus, we recommend investigations on the influence of Mg-based implants on the physis of growing animals.

## Supplementary data

Supplementary data are available at *Regenerative Biomaterials* online.

## Supplementary Material

rbac077_Supplementary_Data
