# Supplementary figures and images for: Degradation behavior and osseointegration of Mg–Zn–Ca screws in different bone regions of growing sheep: a pilot study
Source: Regen Biomater. 2022 Oct 18;10:rbac077. doi: 10.1093/rb/rbac077 (PMC9845522; doi:10.1093/rb/rbac077)

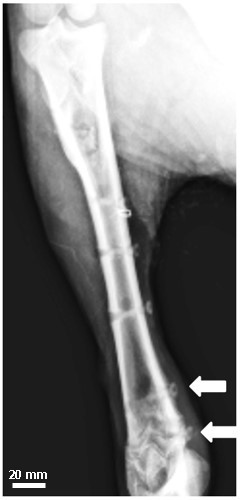

Supplement: rbac077_Supplementary_Data [file rbac077_supplementary_data.zip › rbac077_Supplementary_Data/Suppl. Figure S1.jpg]

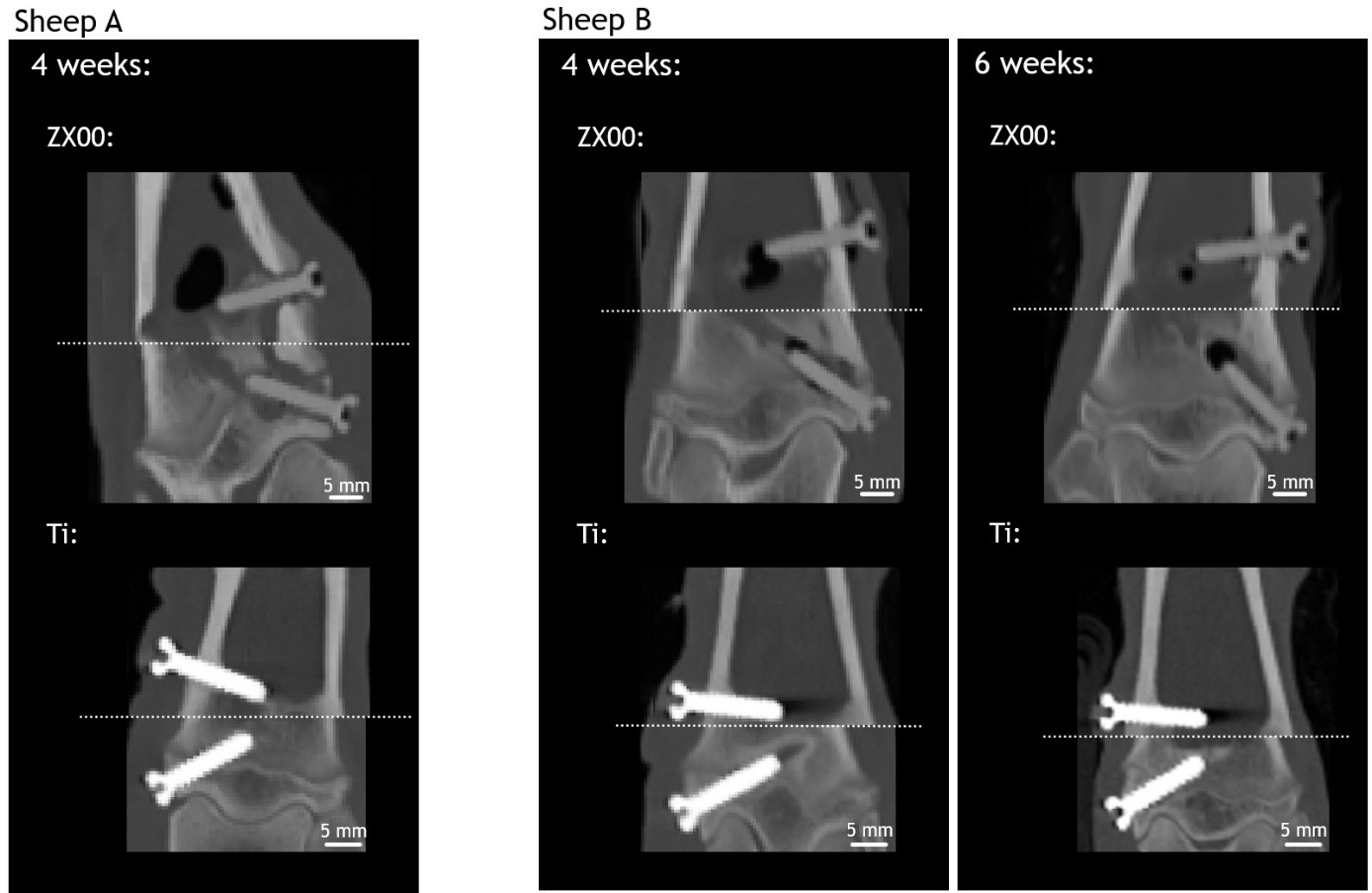

Supplement: rbac077_Supplementary_Data [file rbac077_supplementary_data.zip › rbac077_Supplementary_Data/Suppl. Figure S2.jpg]

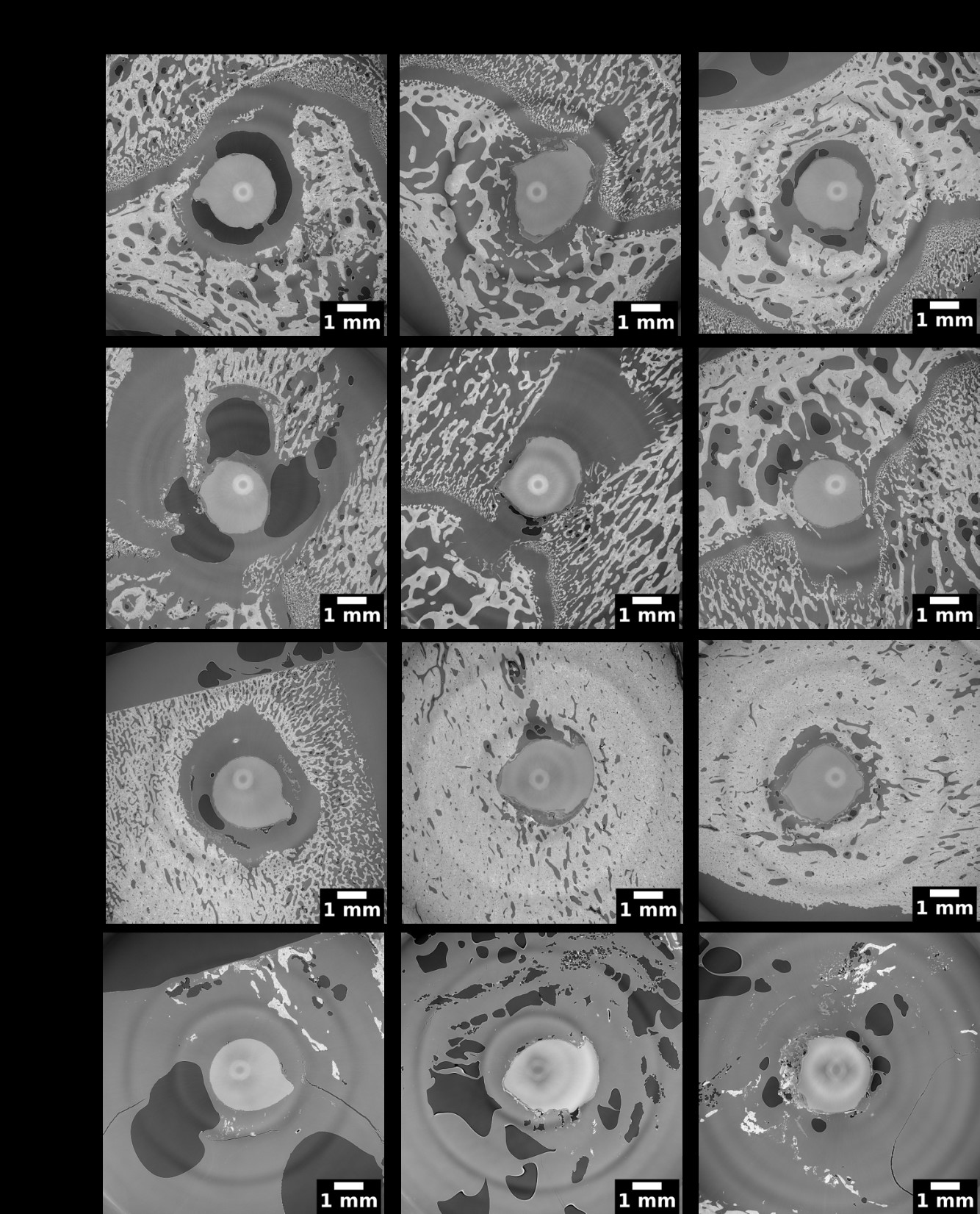

Supplement: rbac077_Supplementary_Data [file rbac077_supplementary_data.zip › rbac077_Supplementary_Data/Suppl. Figure S3.jpg]

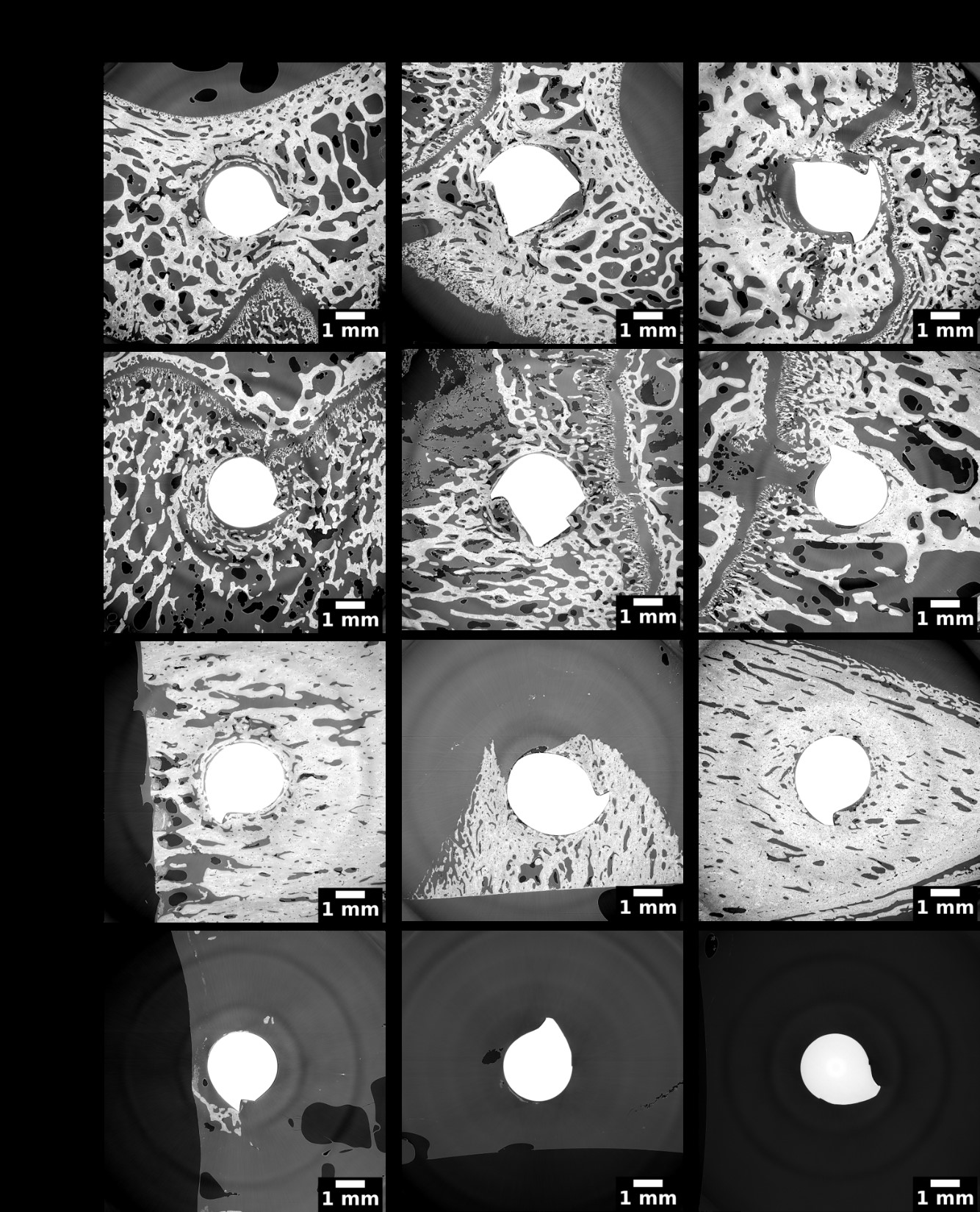

Supplement: rbac077_Supplementary_Data [file rbac077_supplementary_data.zip › rbac077_Supplementary_Data/Suppl. Figure S4.jpg]

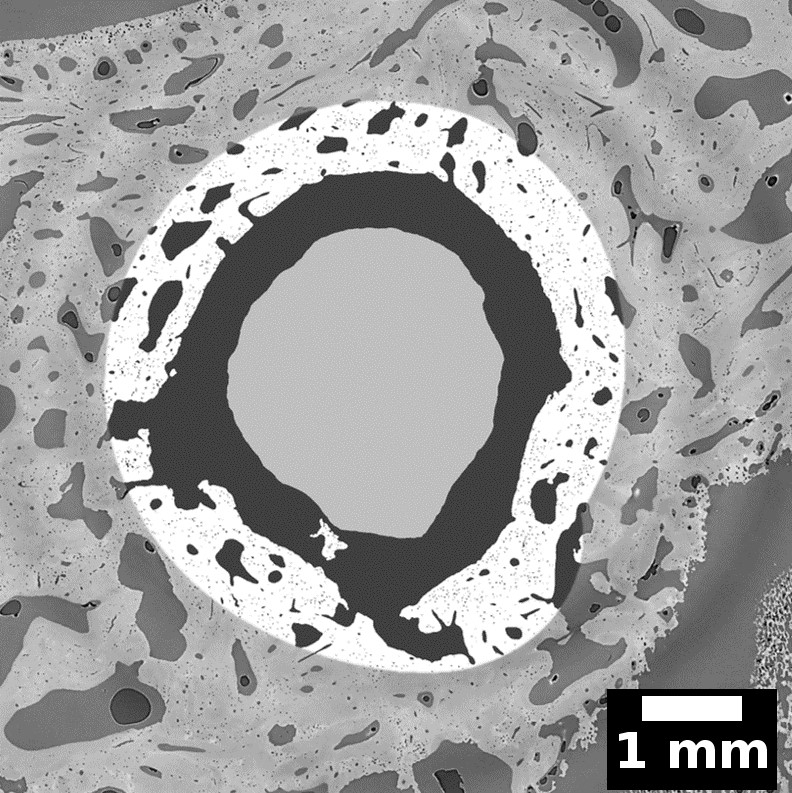

Supplement: rbac077_Supplementary_Data [file rbac077_supplementary_data.zip › rbac077_Supplementary_Data/Suppl. Figure S5.jpg]

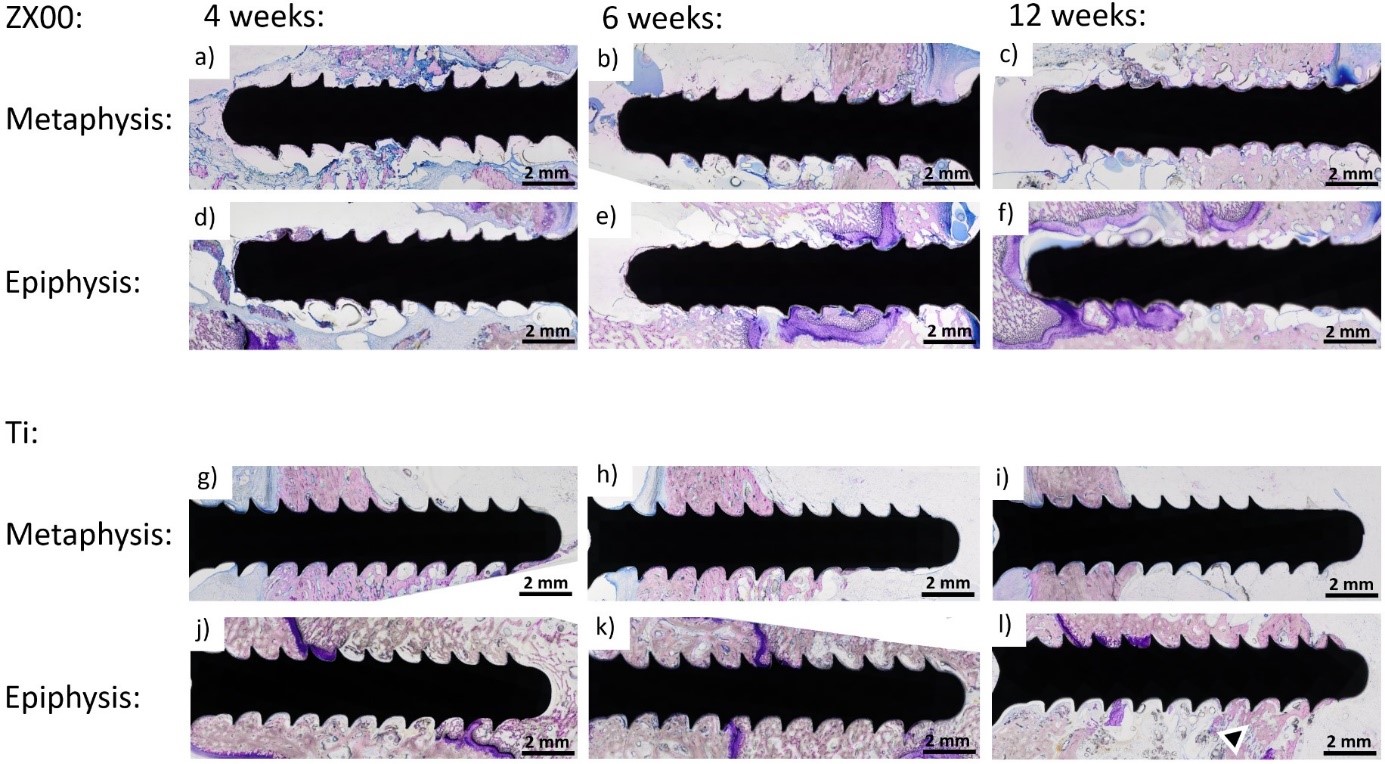

Supplement: rbac077_Supplementary_Data [file rbac077_supplementary_data.zip › rbac077_Supplementary_Data/Suppl. Figure S6.jpg]

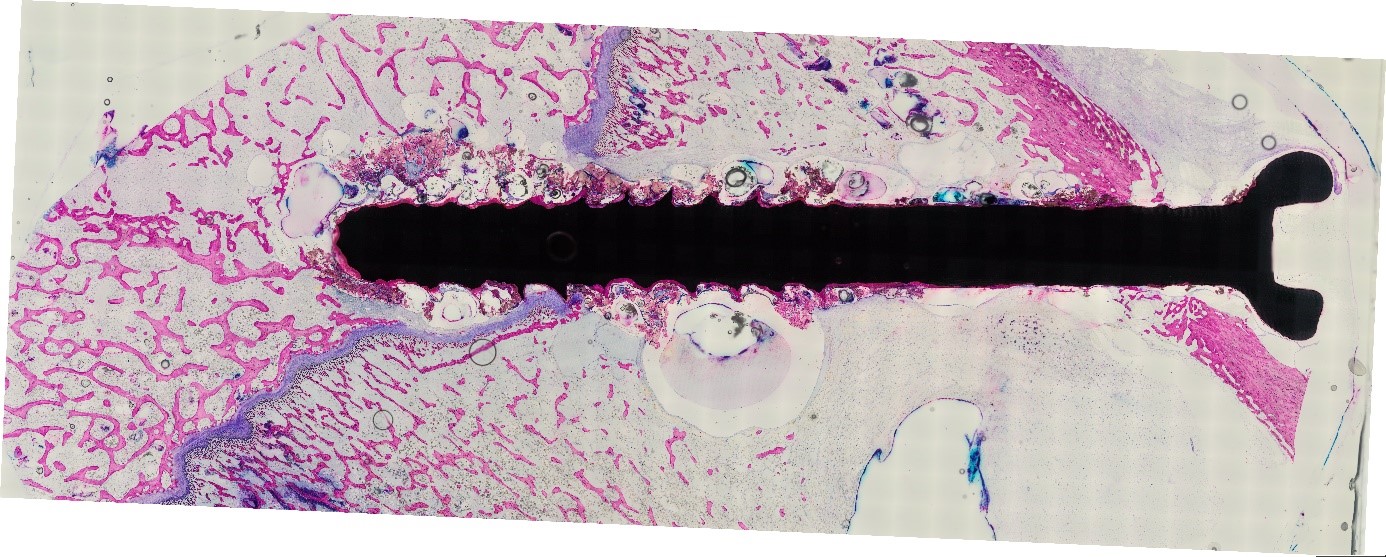

Supplement: rbac077_Supplementary_Data [file rbac077_supplementary_data.zip › rbac077_Supplementary_Data/Suppl. Figure S7.jpg]
