# Supplementary material for: Degradation behavior and osseointegration of Mg–Zn–Ca screws in different bone regions of growing sheep: a pilot study
Source: Regen Biomater. 2022 Oct 18;10:rbac077. doi: 10.1093/rb/rbac077 (PMC9845522; doi:10.1093/rb/rbac077)
Supplement: rbac077_Supplementary_Data [file Revision letter - Regenerative Biomaterials.pdf › rbac077_Supplementary_Data/Revision letter - Regenerative Biomaterials.pdf]

Nicole Gabriele Sommer, PhD
Department of Orthopedics and Traumatology
Medical University of Graz
Auenbruggerplatz 5
A-8036 Graz, Austria
Phone: 0043 316 38578040
Email: nicole.sommer@medunigraz.at

08.09.2022

Dear Editor,

We would like to thank the reviewers for their time and comments on our submitted manuscript entitled "**Degradation behavior and osseointegration of Mg-Zn-Ca screws in different bone regions of growing sheep – a pilot study**" by Romy Marek & Hanna Cwieka et al. We have edited the manuscript to address the reviewer's concerns and attached you will find the point-to-point revision. We hope that the manuscript is now suitable for publication.

Thank you in advance.

Yours sincerely,

……………………………………

Signature and Title (Nicole Gabriele Sommer, PhD)
