# Supplementary material for: Degradation behavior and osseointegration of Mg–Zn–Ca screws in different bone regions of growing sheep: a pilot study
Source: Regen Biomater. 2022 Oct 18;10:rbac077. doi: 10.1093/rb/rbac077 (PMC9845522; doi:10.1093/rb/rbac077)
Supplement: rbac077_Supplementary_Data [file rbac077_supplementary_data.zip › rbac077_Supplementary_Data/Point-to-point revision - Regenerative Biomaterials.docx]

**Point-to-point revision**

1. **Editor’s comments and suggestions:**

Editor: Please notice that the GA should be prepared and displayed at the actual size of 1.4 inches (height) × 3.3 inches (width) or 3.6 cm × 8.4 cm, and it will be presented following the Abstract. Please do not enlarge the GA, because the image will eventually appear on the first page of the published PDF file in the specified size above.

*We thank the Editor for this information. We assume that it was given due to the unreadability of the GA at the required size of 3.6 cm x 8.4 cm. Thus, we changed the GA slightly and enlarged the features.*

**Reviewer’s comments and suggestions:**

**Reviewer #1:**
It is an interesting work, with enough findings to support its publication on Regenerative Biomaterials. Minor revision should be done: I cannot find description about Fig.7 in the main text. And in Fig.7, for the dark area which was depicted as "background", it should be replaced by "gascavity" or something else.

*We thank Reviewer #1 for his/her time and important comments concerning our submitted manuscript.*

- *Figure 7, which was mistakenly marked as Figure 6 in the text, was corrected (see page 17 - 18, Line 349 and 354, in blue): “Figure 7 depicts an example of the degraded ZX00 alloy at the 12-week time point, in comparison to the non-degraded, registered and resampled screw whose outline is marked in white..” and “In the magnified section of Figure 7 the corroded metal and degradation layer are visible, as well as the surrounding trabecular bone with blood vessel and osteocyte lacunae”.*
- *Under section 2.5.3. (page 9, line 184 - 186) we defined, that all remaining features not belonging to residual material, degradation layer, or mineralized bone are defined as “background”. For the kind of evaluation executed under this section, distinguishing between these remaining features is not necessary. As these remaining features consist of various compositions (e.g. gas cavities, soft tissue, etc.), we had to find a neutral definition, which applies for all of them. However, we see that defining the residual features in the results part as background without further explanation might be confusing. Therefore, we included a definition for the word “background” also in the beginning of the results part (page 17, line 343 - 346, in blue): “In order to perform the calculations, we distinguished between residual material (non-corroded alloy), degradation layer (corrosion products attached to the residual material), bone (mineralized tissue) and background (all remaining features not assigned to previously mentioned labels).”*

**Reviewer #2:**

This manuscript made a systematic in vivo study on implantation of a simple compositional magnesium alloy, ZX00, at two different sites in sheep tibiae for different times, in comparison with Ti. The results were rich and reliable by using different analysis techniques, including degradation rate and gas formation, BV and BIC around the implant, and so on, which are valuable for bone repair applications of biodegradable magnesium alloys. Since the study concluded that the degradation rate of ZX00-implants ranged between 0.22-0.75 mm/year, it would be better to further analyze and discuss in the manuscript to see if such degradation rate is suitable for bone repair and healing with taking the effect on the mechanical loading ability of the implant into account.

*We thank Reviewer #2 for this valuable comment, addressing an important issue. The suitability of an implant material for bone repair applications is affected by the DR, but also by the BIC, especially regarding the ability to transfer mechanical load. Therefore, we discussed this topic in several sections in the manuscript.*

- *Page 25, line 511-516, in blue: “Therefore, the screws were exposed to similar tissue types, allowing a comparison between the studies. The DRs calculated for both implantation sites accounted 0.22 – 0.75 mm/y. This lies within the range of DRs of different Mg alloys found in the literature [30,31]. Chaya et al. stated that they achieved fracture healing with DRs of 0.4 mm/year, using pure Mg implants in a rabbit model [32], indicating suitability for bone repair applications. Further, we calculated the DRs from the values found in the work of Holweg et al. [8], using the equation defined under section* ***Fehler! Verweisquelle konnte nicht gefunden werden.****. According to the numbers found in their work, the DRs ranged from 0.21 – 0.83 mm/y, which was sufficient for fracture healing in a sheep model, using ZX00 implants. Here, the fastest DR for the ZX00-screws was found 4 weeks after implantation in both, epiphysis and metaphysis.”*
- *Page 27, line 550-559, in blue: “In order to suffice as implant material for bone repair applications, the DR must be slow enough for the bone to allow gradual replacement of the degraded regions by tissue, to guarantee stability. Additionally, direct contact between the implant and the tissue is necessary, in order to take over some of the mechanical load occurring in the bone until fracture consolidation. The implant is stabilized both by calcified bone, as well as cartilage tissue, which typically form during callus formation [36]. Here, direct BIC was found around both, ZX00 and Ti-screws. For both materials the minimum and maximum values changed between the bone types over time. Thus, we did not find a clear trend for the BIC between the implantation sites and bone types for either material. Yet, the fact that we found comparable BIC for Mg and Ti suggests ability of the bone to form sufficient contact for load distribution during the healing process, which suggests suitability for bone repair applications.”*
- *Page 29, line 595 – 602, in blue: “Besides accelerating the DR, acidosis also inhibits osteoblastic functions and accelerates osteoclastic activity in vitro [49], thus decreasing bone ingrowth, which could be an explanation for the poor BIC at this time point. However, it must be considered that in case of a strong inflammatory reaction or an infection a similar effect is seen also around other materials, such as Ti or PEEK [50,51] and is therefore rather caused by this reaction than the material itself. The poor BIC could impede distribution of mechanical load within the bone at this time point. Nevertheless, only movement stability and not load stability is necessary in case of this surgery technique, reducing the need for a high contact.* *Furthermore, to ensure that infections are not regularly associated with Mg implantation, one additional ZX00-screw from a different animal at the same time point was investigated.”*
